# Immunohistochemical Analysis of Cerebral Thrombi Retrieved by Mechanical Thrombectomy from Patients with Acute Ischemic Stroke

**DOI:** 10.3390/ijms17030298

**Published:** 2016-02-26

**Authors:** Michael K. Schuhmann, Ignaz Gunreben, Christoph Kleinschnitz, Peter Kraft

**Affiliations:** Department of Neurology, University Hospital Würzburg, 97080 Würzburg, Germany; schuhmann_m@ukw.de (M.K.S.); Gunreben_i@ukw.de (I.G.); kleinschni_c@ukw.de (C.K.)

**Keywords:** immune cells, lymphocytes, mechanical thrombectomy, ischemic stroke, inflammation, thrombus formation

## Abstract

Mechanical thrombectomy is a novel treatment option for patients with acute ischemic stroke (AIS). Only a few studies have previously suggested strategies to categorize retrieved clots according to their histologic composition. However, these reports did not analyze potential biomarkers that are of importance in stroke-related inflammation. We therefore histopathologically investigated 37 intracerebral thrombi mechanically retrieved from patients with AIS, and focused on the composition of immune cells and platelets. We also conducted correlation analyses of distinctive morphologic patterns (erythrocytic, serpentine, layered, red, white, mixed appearance) with clinical parameters. Most T cells and monocytes were detected in erythrocytic and red clots, in which the distribution of these cells was random. In contrast, von Willebrand factor (vWF)-positive areas co-localized with regions of fibrin and collagen. While clots with huge amounts of vWF seem to be associated with a high National Institute of Health Stroke Scale score at admission, histologic findings could not predict the clinical outcome at discharge. In summary, we provide the first histologic description of mechanically retrieved intracerebral thrombi regarding biomarkers relevant for inflammation in ischemic stroke.

## 1. Introduction

After successful clinical trials in patients with occlusion of a major intracranial artery, mechanical thrombectomy (MT) with stent retrievers adds a novel therapeutic option in patients with acute ischemic stroke (AIS) [1,2,3]. As MT is only applicable in approximately 5%–10% of patients with ischemic stroke, intravenous thrombolysis (IVT) using recombinant tissue plasminogen activator is still the treatment of choice for most patients with AIS [4]. In IVT [5], but not MT with stent retrievers [6], the response to therapy regarding recanalization and subsequent clinical outcome depends on thrombus length. In contrast, recanalization rates and clinical outcomes are dependent on thrombus density in computed tomography (CT) imaging—and consequently dependent on histologic thrombus composition—in both therapeutic strategies, IVT [7,8] as well as MT [9]. Therefore, knowledge of the detailed clot composition could become helpful to assign patients to a distinct treatment strategy—at the latest when novel treatment options next to IVT and MT arise.

The first histopathologic evaluation of cerebral thrombi was carried out about 50 years ago using post-mortem material [10]. However, a timely assessment of thrombus material immediately after AIS occurrence has only been possible since the development of MT devices a few years ago. Since then, several researchers analyzed the histologic composition of retrieved cerebral clots and suggested different strategies for categorization [11,12,13,14,15]. In addition, an increasing number of imaging studies have been published trying to visualize clot composition with CT [16,17,18] and magnetic resonance imaging (MRI) [13,14,19]. A series of studies evaluating novel MRI contrast agents specific for activated platelets [20] or fibrin [21,22] have been published. In the near future, these imaging strategies could allow identification of individual patients who might profit the most from a therapeutic intervention in AIS, and—*vice versa*—of patients who would most likely not profit from a particular treatment strategy. 

The important role of immune cells on stroke development [23,24,25,26] and the underlying mechanism of thromboinflammation [27] is well established in animal models of ischemic stroke. It has been shown that immune cells (e.g., cluster of differentiation (CD) 4^+^ T cells, CD68^+^ monocytes) interact with molecules that are of importance for platelet signaling (e.g., von Willebrand factor (vWF)) and contribute to thrombus formation [27]. Monocyte–platelet aggregates are increased in patients with acute thrombotic events [28,29]. A recent clinical trial provided the first evidence that a pharmacologically induced lymphocytopenia is also associated with beneficial effects in human AIS [30]. Further clinical stroke studies addressing immunologic targets are on the way (e.g., ClinicalTrials.gov: NCT01955707). 

Until now, the published reports about the histologic characterization of intracranial thrombi mainly focused on coagulation, and a precise assessment of inflammation has not yet been reported. Therefore, we aimed for a detailed characterization of the retrieved cerebral thrombi regarding biomarkers that play major roles in stroke-related inflammation.

## 2. Results

### 2.1. Demographic and Clinical Characterization of Patients

We histologically analyzed 37 thrombi (retrieved between 2012 and 2015) from patients with AIS with a mean age of 66 ± 16 years. Forty-nine percent of patients were male. Twenty-six patients (70%) received IVT before clot retrieval. The mean National Institutes of Health Stroke Scale (NIHSS) score [31] was 17 ± 7 at admission and 7 ± 4 at discharge of the patients. Location of the vessel occlusion was the middle cerebral artery (MCA) in 22 cases, intracranial part of the internal carotid artery (“carotid-T”, C-T) in 10 cases, or the basilar artery (BA) in five cases (Table 1). 

### 2.2. Categorization of Retrieved Clots

**Figure 1:**
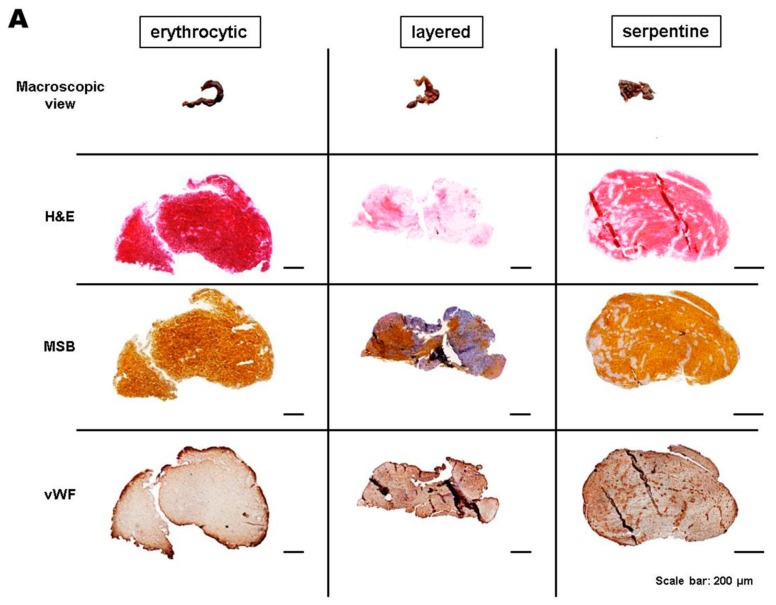

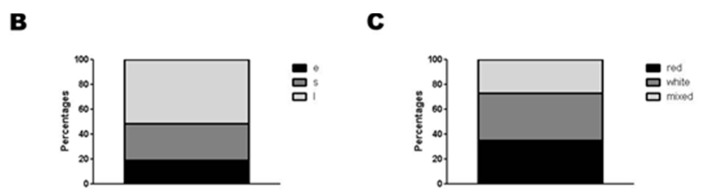
(**A**) Macroscopic view, hematoxylin and eosin (H&E), Martius scarlet blue (MSB), and von Willebrand factor (vWF) staining of three representative thrombi that show erythrocytic, layered, or serpentine morphology; (**B**,**C**) Bar graph of all thrombi after categorization by morphologic subtypes. e, erythrocytic; s, serpentine; l, layered.

After clinical characterization of patients, we histopathologically categorized the extracted thrombi according to distinctive patterns into erythrocytic (19%), layered (30%), and serpentine (51%) [11], and according to the content of the red blood cells (RBCs) and fibrin/collagen as red (35%), white (38%), or mixed (27%) [12] (Table 1, Figure 1A).

**Figure 2:**
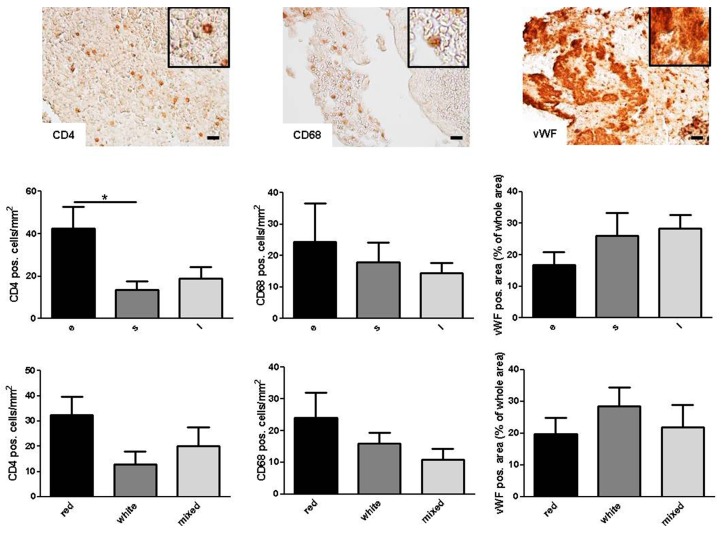
Histologic quantification of T cells (CD4^+^), monocytes (CD68^+^), and platelets (vWF^+^) in all thrombi by morphologic subgroups. e, erythrocytic; s, serpentine; l, layered. Scale bar: 20 µm. Magnification: 20-fold. Inserts show high magnification of representative staining. * *p* < 0.05.

In the next step, we correlated the histopathologic thrombus subgroups (Figure 1B,C) with the number of immune cells (CD4^+^ T cells, CD68^+^ monocytes) and the fraction of vWF^+^ areas. Importantly, a significant accumulation of CD4^+^ T cells (*p* < 0.05) and a trend for CD68^+^ monocytes (*p* > 0.05) was detected in erythrocytic when compared with serpentine thrombi (Figure 2). The CD4^+^ and CD68^+^ cells were randomly distributed within the clot. In contrast, white thrombi (79% layered and 21% serpentine) showed higher percentages of vWF^+^ areas that are co-localized with the regions of fibrin/collagen (*p* > 0.05) (Figure 2).

### 2.3. Correlation of Histologic Results with Clinical Parameters

**Figure 3:**
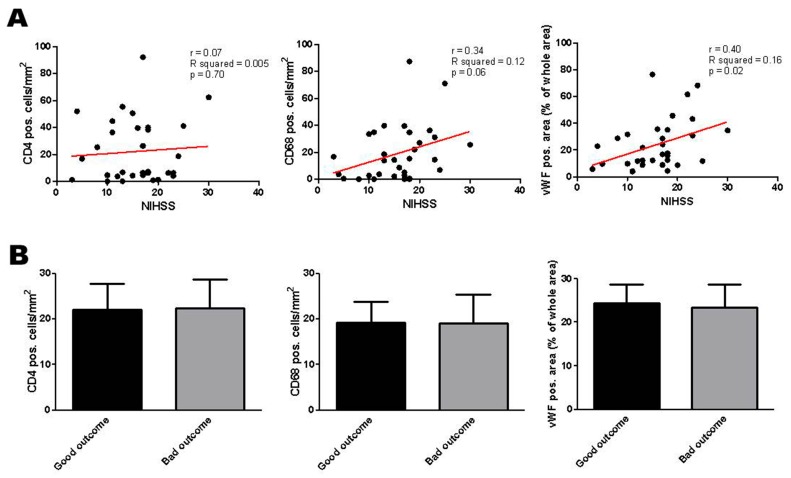
(**A**) Cellular thrombus histology of all retrieved clots except from the basilar artery in correlation to NIHSS scores at admission and clinical outcome at discharge. Red line: linear regression curve; (**B**) Categorization of CD4^+^ T cells, CD68^+^ monocytes and vWF^+^ platelets into “Good outcome” (NIHSS score 0–4 or improvement >9 points) or “Bad outcome” at the time of discharge. NIHSS, National Institute of Health Stroke Scale. r = correlation coefficient. R squared = coefficient of determination. p = level of significance.

Finally, linear regression analysis was performed to correlate the number of immune cells or vWF^+^ areas within the thrombi with the NIHSS score as a marker for AIS symptom severity. The number of CD68^+^ monocytes and vWF^+^ platelets—but hardly the number of CD4^+^ T cells—displayed a clear trend to correlate with a high NIHSS score at admission (Figure S1). To reduce sample inhomogenities, we statistically re-evaluated our findings after exclusion of basilar artery occlusions. Here, the associations between CD68^+^ monocytes (*p* = 0.06) or vWF^+^ platelets (*p* = 0.02) and NIHSS score were even higher (Figure 3). In contrast, neither the number of CD4^+^ and CD68^+^ nor the area of vWF^+^ cells was predictive of the clinical outcome at discharge (Figure 3B). In a multivariate linear regression model, neither age nor sex significantly influenced these results (data not shown). Also Trial of Org 10172 in Acute Stroke Treatment (TOAST) [32] criteria and thrombolysis in cerebral infarction (TICI) scores [33] (Table S1) had no impact on the fraction or distribution of immune cells or platelets in the clots (data not shown).

## 3. Discussion

In this study, we analyzed the histologic composition of cerebral clots retrieved by MT from patients with AIS. In contrast to previous publications [11,12,13,14], we here, for the first time, provide a detailed characterization of clots regarding the number and distribution of distinct immune cells known to play major roles in inflammatory mechanisms of experimental ischemic stroke in rodents [24,25,26,27,34] and which could also be highly relevant for the pathophysiology of human AIS. 

The baseline categorization of cerebral thrombi regarding distinctive patterns (erythrocytic, layered, or serpentine) and the content of RBCs and fibrin/collagen (red, white, or mixed) is based on previous reports [11,12]. In contrast, the immunohistologic characterization of CD4^+^ T cells, CD68^+^ monocytes and vWF^+^ cells (considered to be platelets) adds valuable new pathomechanistic information for the understanding of the composition of cerebral clots in special and thrombus formation in the context of human AIS in general. 

Immunohistologic work-up of the thrombi revealed that the number of CD4^+^ T cells and CD68^+^ monocytes was increased in erythrocytic and red clots compared with the other morphologic groups. In contrast, white thrombi comprised more vWF^+^ cells compared with red and mixed clots. Given the promising results of studies that tried to visualize cerebral clot composition using CT and MRI imaging [12,13], it might be feasible to indirectly predict immunologic clot composition by these routine imaging techniques. Moreover, the development of specific MRI contrast agents for immune cells [35] and platelets [36] could further pave the way for non-invasive, but sensitive and specific, novel imaging possibilities for *in vivo* characterization of intracranial clot composition regarding thromboinflammation.

There is increasing evidence that immune cells are not only biomarkers after AIS [37], but also potential therapeutic targets [38]. Nevertheless, the mechanisms by which immune cells contribute to AIS pathophysiology have not been understood in depth so far. Even though we did not observe unambiguous platelet-monocyte co-localizations within the retrieved clots, it appears plausible that the interaction of immune cells with platelets (e.g., via cluster of differentiation 40 (CD40) and CD40 ligand or P-selectin (CD62-P) and P-selectin glycoprotein ligand 1) [27,39] and endothelial cells (e.g., via intercellular adhesion molecule 1 and lymphocyte function-associated antigen 1) [24] also plays a role in human AIS. Based on this hypothesis, formation of platelet–leukocyte aggregates and leukocyte activation might not only contribute to vascular repair, but also to thrombus formation leading to AIS. While most of the preclinical studies dealing with the role of immune cells in AIS development focused on microcirculatory dysfunction [26] or pathomechanisms within the brain [40], the intracranial clots retrieved by MT reflect thrombus formation outside the brain, as most of the patients suffered AIS due to a cardioembolic or arterioarterial embolic event (see TOAST classification in Table S1). Nevertheless, also in the pathogenesis of peripheral (*i.e.*, outside the central nervous system) macroangiopathic atherosclerosis, various immune cells are involved [41,42,43] and there is ongoing effort for *in vivo* visualization of atherosclerotic plaque composition [44,45]. Importantly, and in contrast to Niesten and co-workers [12] who reported AIS subtype-specific differences regarding the percentage of RBC infiltration, we found no differences in immune cell infiltration within the clots when having a detailed look at AIS subtypes according to TOAST criteria. Further clinical studies are needed to assess whether immune cell composition of clots—measured by non-invasive imaging—could predict the response to revascularization strategies, and, in the future, may influence the decision of which treatment option is most suitable in individual cases.

There are several limitations of this study that must be considered. First, the number of patients that could be recruited and consequently the power of statistical analyses are low. Reasons for this are the low number of MTs (only 5%–10% of all patients with AIS) and the difficulties in receiving informed consent from a patient severely affected by AIS. Therefore, patients who have suffered a severe stroke and/or have aphasia could be underrepresented in this study (compared with patients with milder symptoms) because neurologic deficits related to their condition may have prevented them from being capable of providing informed consent; Second, despite a recent publication showing that stent-retriever MT does not lead to significant intimal damage [46], it is possible that the procedure of MT itself might dislocate the original clot composition and thereby could influence the results of our histologic analysis; Furthermore, thrombi retrieved as multiple fragments could not be further processed for immunohistochemistry. Third, it was not possible to conduct a standardized follow-up of patients due to limited patient numbers and low response rates.

## 4. Materials and Methods

### 4.1. Patient Population and Study Design

We histopathologically studied a convenience sample of 37 occluding clots that were mechanically retrieved from large intracranial arteries of patients with AIS at the Department of Neurology, University Hospital of Würzburg, Germany. The study protocol was approved by the ethics committee of the Medical Faculty of the University of Würzburg, Germany (reference number 36/2012) and written informed consent was provided by all participants. Inclusion criteria were: patients with AIS ≥ 18 years with an occlusion of the proximal MCA, the C-T, or the BA, successful MT and informed consent of the patients or their legal representatives during the hospital treatment. The functional status of the patients was assessed using the NIHSS score [31] at hospital admission and again before discharge. Good neurologic outcome was defined as an NIHSS score of 0–4 or an NIHSS score improvement of >9 points [46]. The TICI score [33] was used to assess post-intervention vessel patency and has been performed by an independent investigator (Ignaz Gunreben) blinded to clinical and histologic outcome: (0) no perfusion; (1) penetration with minimal perfusion; (2) partial perfusion (2a, only partial filling (less than two-thirds) of the entire vascular territory is visualized); 2b, complete filling of all of the expected vascular territory is visualized, but the filling is slower than normal; (3) complete perfusion. The TOAST (Trial of Org 10172 in Acute Stroke Treatment) criteria [31] were applied to describe the assumed etiology of AIS: (1) cardioembolism; (2) large-artery atherosclerosis; (3) small-vessel occlusion; (4) other determined etiology; or (5) undetermined etiology.

### 4.2. Thrombectomy Procedure

MT was performed in accordance with local standard operating procedures and common recommendations [47]. Only stent retrievers were used. The MT was performed under general anesthesia in all of the patients. Participation in the study had no impact on the way the patients were treated.

### 4.3. Processing of Thrombi and Analysis

Immediately after clot retrieval, *i.e.*, still within the catheter laboratory, thrombus material was fixed in phosphate-buffered formalin. The formalin-fixed specimens were embedded in paraffin (Leica, Wetzlar, Germany), cross-sectioned at 4-µm thickness and stained with hematoxylin and eosin (H&E), and Martius scarlet blue (MSB) (Atom Scientific, Cheshire, UK). Subsequently, based on H&E staining, thrombi were characterized according to their overall appearance into erythrocytic, layered, and serpentine [11]. Categorization was done by visual assignment of two independent investigators (Michael K. Schuhmann, Peter Kraft). In case of divergent results after first view, investigators independently re-categorized the clots and finally had to reach an agreement. Additionally, using MSB-stained sections, the content of RBCs and fibrin/collagen was quantitatively determined. Accordingly, thrombi were classified as *red* (RBCs outnumber fibrin/collagen ≥ 15%) or *white* (fibrin/collagen outnumber RBCs ≥ 15%). All others were classified as *mixed* [12]. To assess for thromboinflammation, all thrombi were stained immunohistochemically for CD4^+^ T cells (abcam; ab133616, Cambridge, UK), CD68^+^ monocytes (Acris; AM331235U-N, Herford, Germany) and von Willebrand factor (abcam; ab6994, Cambridge, UK). 

### 4.4. Statistical Analysis

All results are presented as mean ± standard error of the mean. Distribution of data was evaluated using the Kolmogorov–Smirnov test. To test for significant differences between multiple groups, one-way analysis of variance with *post hoc* Bonferroni adjustment for *p*-values was used. The Pearson test was used to analyze the correlation between the number of immune cells or platelets and NIHSS at admission. To rule out potential confounders, we utilized a multivariate linear regression model adjusted for age and sex. In a second multivariate linear regression model we evaluated for TICI score and TOAST classification. *p-*values <0.05 were considered significant with * *p* < 0.05.

## 5. Conclusions

Intracranial thrombi retrieved by MT from patients with AIS comprise T cells, monocytes, and platelets as cellular components and potential inflammatory biomarkers that might also be relevant in the pathophysiology of human AIS. Our findings should stimulate further investigations to identify whether the immune cell composition of clots may influence the clinical response to IVT or MT.

## Figures and Tables

**Table 1:** Clinical characteristics of patients and categorization of thrombus histology.

No.	Sex	Age, Years	Smoker	Vascular Site	Lysis	NIHSS Admission	NIHSS Discharge	Thrombus Histology
1	F	54	Yes	Right MCA	Yes	15	8	e/red
2	F	72	No	Right C-T	No	4	8	e/red
3	F	83	No	Right C-T	Yes	17	7	e/red
4	F	57	No	Left MCA	Yes	18	2	s/red
5	M	61	Yes	Left MCA	Yes	16	4	l/mixed
6	M	42	No	Left C-T	Yes	18	10	l/white
7	M	50	Yes	Right MCA	Yes	24	3	s/mixed
8	F	72	No	BA	No	23	9	l/red
9	F	74	No	BA	Yes	28	10	l/red
10	F	59	No	Left MCA	No	23	7	s/white
11	F	80	No	Left C-T	Yes	23	14	l/white
12	M	56	Yes	Right MCA	Yes	8	8	e/red
13	M	54	No	Right MCA	Yes	30	11	l/white
14	F	83	Yes	BA	Yes	31	Deceased	s/mixed
15	F	56	No	Left MCA	Yes	17	10	l/mixed
16	M	58	No	Left MCA	Yes	18	8	s/red
17	M	57	No	Left MCA	Yes	10	4	l/white
18	F	84	No	Left MCA	Yes	15	9	s/white
19	F	75	No	Left C-T	Yes	19	7	l/mixed
20	M	61	Yes	BA	Yes	4	0	l/white
21	M	67	No	Left C-T	No	13	15	l/white
22	M	75	No	Left MCA	Yes	13	3	l/white
23	F	82	No	Right MCA	Yes	22	5	l/white
24	F	83	No	Right MCA	No	17	7	e/red
25	F	80	No	Right MCA	Yes	5	2	s/mixed
26	F	69	No	Left MCA	Yes	18	13	e/red
27	M	43	No	Left MCA	Yes	18	6	s/red
28	F	93	No	Right MCA	Yes	11	4	l/white
29	M	40	No	Right MCA	Yes	10	1	l/white
30	M	77	No	Left C-T	Yes	20	13	s/white
31	M	28	No	Right C-T	Yes	12	0	s/mixed
32	F	63	No	Left MCA	No	25	10	s/red
33	F	84	No	Right MCA	No	17	9	l/white
34	M	47	No	Right C-T	No	11	1	e/red
35	M	95	n.d.	Left MCA	No	13	Deceased	l/red
36	M	58	Yes	Left C-T	No	3	12	l/mixed
37	M	60	Yes	BA	No	23	3	l/mixed

BA, basilar artery; C-T, intracranial part of the internal carotid artery (“carotid-T”); F, female; M, male; MCA, middle cerebral artery; thrombus histology: e, erythrocytic; l, layered; s, serpentine.

## Supplementary Materials

Supplementary materials can be found at http://www.mdpi.com/1422-0067/17/3/298/s1.

## Abbreviations

AIS: acute ischemic stroke
BA: basilar artery
CD: cluster of differentiation
C-T: intracranial part of the internal carotid artery
CT: computed tomography
H&E: hematoxylin and eosin
IVT: intravenous thrombolysis
MCA: middle cerebral artery
MRI: magnetic resonance imaging
MSB: Martius scarlet blue
MT: mechanical thrombectomy
NIHSS: National Institutes of Health Stroke Scale
RBC: red blood cell
TOAST: Trial of Org 10172 in Acute Stroke Treatment
TICI: thrombolysis in cerebral infarction
vWF: von Willebrand factor
