# Supplementary material for: Immunohistochemical Analysis of Cerebral Thrombi Retrieved by Mechanical Thrombectomy from Patients with Acute Ischemic Stroke
Source: Int J Mol Sci. 2016 Feb 26;17(3):298. doi: 10.3390/ijms17030298 (PMC4813162; doi:10.3390/ijms17030298)
Supplement: Supplementary file 1 [file ijms-17-00298-s001.pdf]

Supplementary Materials: Immunohistochemical Analysis of Cerebral Thrombi Retrieved by Mechanical Thrombectomy from Patients with Acute Ischemic Stroke

Michael K. Schuhmann, Ignaz Gunreben, Christoph Kleinschnitz and Peter Kraft

Table S1. Clinical characteristics of patients and categorization of thrombus histology.

No.	TOAST	TICI
1	Cardioembolic	3
2	Unknown	2b
3	Cardioembolic	3
4	Cardioembolic	2b
5	Unknown	2a
6	Unknown	3
7	Large-artery atherosclerosis	2b
8	Cardioembolic	2a
9	Cardioembolic	2b
10	Cardioembolic	3
11	Cardioembolic	2a
12	Unknown	2b
13	Unknown	0
14	Unknown	3
15	Cardioembolic	2b
16	Cardioembolic	3
17	Unknown	3
18	Cardioembolic	3
19	Unknown	2a
20	Cardioembolic	3
21	Unknown	0
22	Cardioembolic	2a
23	Cardioembolic	3
24	Cardioembolic	2a
25	Cardioembolic	3
26	Cardioembolic	2a
27	Large-artery atherosclerosis	3
28	Large-artery atherosclerosis	2b
29	Unknown	3
30	Cardioembolic	2a
31	Large-artery atherosclerosis	2b
32	Unknown	2a
33	Cardioembolic	3
34	Large-artery atherosclerosis	2a
35	Unknown	2b
36	Large-artery atherosclerosis	2b
37	Cardioembolic	3

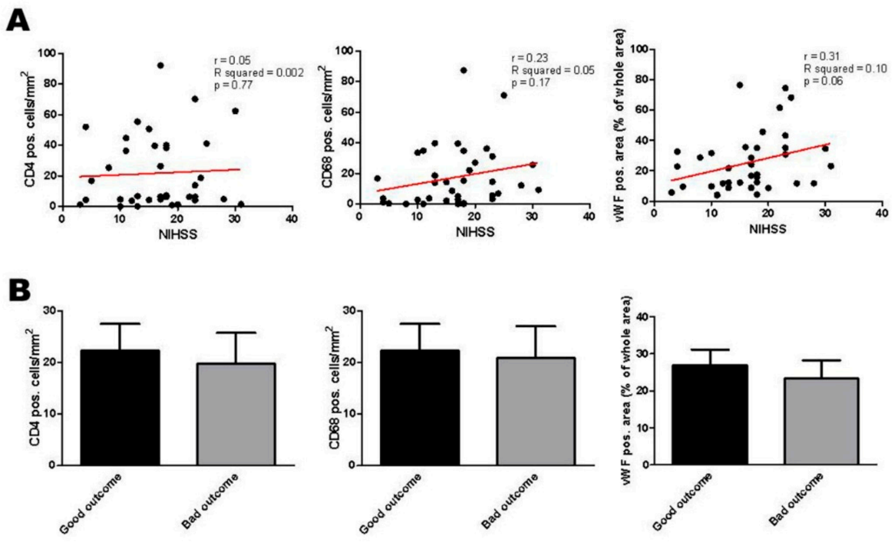

Figure S1. (**A**) Cellular thrombus histology of all retrieved clots in correlation to NIHSS scores at admission and clinical outcome at discharge. Red line: linear regression curve; (**B**) Categorization of $CD4^{+}$ T cells, $CD68^{+}$ monocytes and vWF^{+} platelets into "Good outcome" (NIHSS score 0–4 or improvement >9 points) or "Bad outcome" at the time of discharge. NIHSS, National Institute of Health Stroke Scale.
